# Temporal learning analytics to explore traces of self-regulated learning behaviors and their associations with learning performance, cognitive load, and student engagement in an asynchronous online course

**DOI:** 10.3389/fpsyg.2022.1096337

**Published:** 2023-01-23

**Authors:** Jerry Chih-Yuan Sun, Yiming Liu, Xi Lin, Xiao Hu

**Affiliations:** ^1^Institute of Education, National Yang Ming Chiao Tung University, Hsinchu, Taiwan; ^2^Faculty of Education, The University of Hong Kong, Hong Kong SAR, China; ^3^College of Education, East Carolina University, Greenville, NC, United States

**Keywords:** temporal learning analytics, educational data mining, asynchronous online course, self-regulated learning, cognitive load, student engagement

## Abstract

Self-regulated learning (SRL) plays a critical role in asynchronous online courses. In recent years, attention has been focused on identifying student subgroups with different patterns of online SRL behaviors and comparing their learning performance. However, there is limited research leveraging traces of SRL behaviors to detect student subgroups and examine the subgroup differences in cognitive load and student engagement. The current study tracked the engagement of 101 graduate students with SRL-enabling tools integrated into an asynchronous online course. According to the recorded SRL behaviors, this study identified two distinct student subgroups, using sequence analysis and cluster analysis: high SRL (H-SRL) and low SRL (L-SRL) groups. The H-SRL group showed lower extraneous cognitive load and higher learning performance, germane cognitive load, and cognitive engagement than the L-SRL group did. Additionally, this study articulated and compared temporal patterns of online SRL behaviors between the student subgroups combining lag sequential analysis and epistemic network analysis. The results revealed that both groups followed three phases of self-regulation but performed off-task behaviors. Additionally, the H-SRL group preferred activating mastery learning goals to improve ethical knowledge, whereas the L-SRL group preferred choosing performance-avoidance learning goals to pass the unit tests. The H-SRL group invested more in time management and notetaking, whereas the L-SRL group engaged more in surface learning approaches. This study offers researchers both theoretical and methodological insights. Additionally, our research findings help inform practitioners about how to design and deploy personalized SRL interventions in asynchronous online courses.

## 1. Introduction

As the COVID-19 pandemic continues, there is a recent trend shifting from technology-assisted or blended learning toward totally online learning among universities worldwide (Hew et al., 2020). Online courses are usually provided in two modes: synchronous and asynchronous. Compared with the former, asynchronous online learning (AOL) can hold larger numbers of students, afford greater flexibility in time and space, and encompass greater student autonomy (Yoon et al., 2021). For example, asynchronous online courses (AOCs) enable students to learn anytime and anywhere. This is particularly beneficial to students who face practical challenges managing time zone differences and unstable internet access during the pandemic. Moreover, students can proceed through the course at their own pace, resulting in learner-centered learning processes (Kim et al., 2021). Despite this, students are often confronted with difficulties sustaining commitment in AOCs (Alhazbi and Hasan, 2021). For example, due to the lack of real-time learning support from instructors and peers, online learners struggle to organize and manage their learning tasks by themselves, causing negative learning experiences and outcomes (Seufert, 2020). Therefore, this time-independent delivery mode requires learners to enact self-regulated learning (SRL) strategies to plan and manage their learning processes independently. A review article by Wong et al. (2019a) reveals that considerable efforts have been made to integrate SRL-enabling tools into AOCs to support SRL strategy use. Unfortunately, even when presented with opportunities to facilitate self-regulation in AOL environments, not all students adopted optimal SRL behaviors to achieve expected learning outcomes (Fincham et al., 2018; Wong et al., 2019a). Therefore, it is necessary to (1) identify subgroups of students with different patterns of SRL behaviors and (2) examine subgroup differences regarding learning outcomes.

The person-centered approach is considered suitable because it can identify homogeneous clusters of individuals who exhibit similar features within their cluster but function in a different way compared with those from other clusters (Hong et al., 2020). Previous studies (e.g., Zheng et al., 2019) utilize various person-centered approaches (e.g., cluster analysis) to classify students according to SRL behaviors. However, many of them rely strongly on self-report measures of SRL behaviors, which suffer from issues including response bias and generate limited information about actual SRL strategy use (Baker et al., 2020). Moreover, even in those studies that remove the aforementioned restrictions of self-reports by using behavioral data (e.g., clickstreams), students are profiled based on the cumulative frequencies of SRL behaviors, which ignores the dynamic and contextual nature of SRL (Azevedo, 2014; Siadaty et al., 2016). In other words, the aggregate, nontemporal representations of SRL behaviors fail to retain any information about how students perform SRL over time and how their learning activities are adapted to meet specific task and environmental demands (Baker et al., 2020). Therefore, whether and how chronological representations of SRL behaviors can be used to identify student subgroups warrants investigation.

In recent years, there have been increasing numbers of attempts to compare learning performance across students’ SRL profiles in online learning environments (e.g., Cicchinelli et al., 2018; Lan et al., 2019). However, little is known about the differences in cognitive load (CL) and student engagement (SE) between SRL profiles, especially in the context of AOL. When studying in AOCs, in addition to dealing with the learning task at hand, students have to handle decisions that instructors are often responsible for, including planning how to proceed and reflecting on what they already learned (Seufert, 2018, 2020). Such additional demands require students to exert effective self-regulation, which otherwise might cause “mental fatigue” or cognitive overload that impedes learning (Seufert, 2018). Moreover, recent review studies building bridges between SRL and CL make theoretical arguments that self-regulation of learning processes relates to cognitive load (Seufert, 2018, 2020; de Bruin et al., 2020). Nevertheless, little empirical evidence to date has been found to verify this argument in AOL settings. Additionally, SE is another crucial determinant of online learners’ academic success (Wong and Liem, 2021). When switching to “emergency remote learning” during COVID-19, students found themselves fighting “digital burnout” or “online learning fatigue” and thus disengaged from course activities (Martin et al., 2022). Prior research suggests that students’ SRL strategies, as well as SRL profiles, have associations with their engagement in AOCs (e.g., Anthonysamy et al., 2020; Pérez-Álvarez et al., 2020). However, to our knowledge, no study exists to investigate how actual behavioral processes of SRL relate to SE in AOL environments. Therefore, whether and how subgroups of students with distinct patterns of SRL behavioral trajectories differ in CL and SE warrants investigation.

The emergence of temporal learning analytics allows researchers to explore whether student subgroups can be identified based on temporal SRL behaviors, compare how SRL behaviors of student subgroups act dynamically over time, and interpret why student subgroups differ in learning outcomes (Knight et al., 2017; Chen et al., 2018; Saint et al., 2022). In temporal learning analytics, two common types of temporal features are considered: the passage of time (e.g., how much time learners spend on learning tasks) and the temporal order (e.g., how events or states are sequentially organized; Chen et al., 2018). The current study focused on analyzing the temporal order of SRL behaviors. Although increasing studies have taken the temporality of SRL into account, SRL researchers (e.g., Saint et al., 2020a,b) point out that most temporal analyses of SRL lack sound theoretical underpinning or use a single analytical method, raising the concerns of ontologically flat explanations of learning as proposed by Reimann et al. (2014). This study captured students’ SRL behaviors as they interacted with SRL-enabling tools embedded in an AOC designed based on Zimmerman (2000) three-phase model and Barnard et al. (2009) online SRL strategies. Then, we combined lag sequential analysis and epistemic network analysis to articulate and compare patterns of how students’ SRL behaviors unfold throughout the course. Such a combination can significantly enhance our understanding of the temporal nature of the SRL processes.

### 1.1. Temporal learning analytics for SRL in AOL environments

SRL refers to “an active, constructive process whereby learners set goals for their learning and then attempt to monitor, regulate, and control their cognition, motivation, and behavior, guided and constrained by their goals and the contextual features of the environment” (Pintrich, 2000, p: 453). In developing various SRL models, researchers have reached a consensus that SRL is a cyclical and dynamic process (Panadero, 2017). Zimmerman (2000) divides SRL processes into three cyclical phases: forethought, performance, and self-reflection, each containing specific SRL strategies that learners are expected to execute. Furthermore, researchers increasingly emphasize SRL as highly context-specific due to continuous innovation in learning formats (Kim et al., 2018). To capture and measure the essence of online SRL, Barnard et al. (2009) operationalized the three-phase model by conceptualizing six constructs: goal setting, environmental structuring, task strategies, time management, help seeking, and self-evaluation. Based on these online SRL constructs, many studies have captured actual online SRL behaviors (e.g., Ye and Pennisi, 2022) and perceived online SRL strategies (e.g., Sun et al., 2017) and have developed interventions for promoting online SRL (e.g., Lai et al., 2018). However, these studies have paid little attention to the temporal dynamics of these online SRL behaviors.

Advances in SRL theory, learning technology, and analytic method have motivated the emergence of temporal learning analytics for SRL (Knight et al., 2017; Chen et al., 2018). First, modern SRL research conceptualizes SRL as a series of temporal events that learners perform during actual learning situations rather than as stable and decontextualized traits or aptitudes (Winne, 2010; Azevedo, 2014). Second, advanced learning technologies (e.g., intelligent tutoring systems) have been developed for tracing temporal characteristics of SRL by recording fine-grained behavioral data on the fly (Azevedo et al., 2018; Azevedo and Gašević, 2019). Third, recent developments in temporal analysis methods have further spurred researchers to undertake temporal analyses of SRL (see review by Saint et al., 2022).

By reviewing existing empirical studies employing behavioral data to explore the temporal dynamics of self-regulation in AOL, we found that very few studies (e.g., Cicchinelli et al., 2018; Fincham et al., 2018; Srivastava et al., 2022) have attempted to identify student subgroups by comparing SRL traces across individual students. For example, based on traces of SRL activities codified from log files captured by learning management systems (LMSs), Cicchinelli et al. (2018) divided learners into four subgroups (i.e., continuously active, inactive, procrastinators, and probers) utilizing sequence analysis and agglomerative hierarchical clustering. Additionally, the majority of relevant studies reveal and compare processes or patterns in online SRL by student subgroups using various temporal analytical techniques including, but not limited to: lag sequential analysis (LSA), epistemic network analysis (ENA), process mining (PM), and sequential pattern mining (SPM; e.g., Saint et al., 2020a; Hwang et al., 2021; Zheng et al., 2021; Sun et al., 2023). For example, Wong et al. (2019b) leveraged SPM to explore 103 Massive Open Online Course (MOOC) learners’ interactive sequences with course activities related to SRL and compared the differences in sequential patterns between students who viewed the SRL-prompt videos and those who did not.

In sum, researchers have illustrated heterogeneity in student SRL behaviors in AOL environments. However, most of them established student subgroups based on (quasi-) experimental designs or through comparisons of cumulative counts of SRL behaviors across students. The use of temporal SRL behaviors for detecting student subgroups is still an underexplored area of research but is one that can extend our current knowledge on the complex nature of temporally unfolding SRL processes. Additionally, although many temporal analyses were undertaken using the same data source in similar learning contexts, their research findings are not entirely consistent and may even be contradictory. One reason for this is that these researchers generally adopt a single analytical approach per study, and different analytical approaches between studies may lead to inconsistent research results (Saint et al., 2020a). As Reimann (2009) pointed out, analyses using a single analytical approach may suffer from ontological flatness. Therefore, multiple analytical approaches should be consolidated to confirm and complement each other in examinations of temporal dynamics of SRL.

### 1.2. SRL processes, cognitive load, and student engagement

Cognitive load theory assumes that (1) for learning to take place, information must be encoded into long-term memory by working memory (WM) and (2) human WM is limited in both capacity and duration (Sweller et al., 1998, 2019). When performing complex or novel learning tasks, learners must process large amounts of information and interactions simultaneously, which may overload their finite WM and thus impair academic performance (Sweller, 2010). Sweller et al. (1998) defined cognitive load (CL) as the amount of WM resources required to process complex or novel information. They recognize three types of cognitive load: intrinsic, extraneous, and germane. Intrinsic cognitive load (ICL) refers to the processing resources associated with the inherent properties of the task and is determined by task complexity and learner expertise (Sweller et al., 1998). Extraneous cognitive load (ECL) arises from unnecessary and irrelevant information imposing processing demands due to suboptimal instructional design (Sweller et al., 1998). ECL could distract learners from the task at hand and hamper learning (Stiller and Bachmaier, 2018). Germane cognitive load (GCL) refers to the WM resources that learners devote to dealing with ICL (Sweller et al., 1998). Unlike the other two loads, GCL helps with schema construction and automation and thus benefits learning (Miller et al., 2021). Appropriate instructional design can manage ICL, reduce ECL, and encourage GCL while still preventing overload (Van Merriënboer and Sweller, 2010).

Researchers have recently extended previous research on CL by unraveling the intricate relationship between SRL and CL (de Bruin et al., 2020; Seufert, 2020). Eitel et al. (2020) propose that (1) CL results not only from how instruction is designed but also from how learners process this instruction and (2) how instruction is processed by learners depends on their ability and willingness to exert self-control. According to Baumeister et al. (2007), self-control is portrayed as a conscious, deliberate, and effortful subset of self-regulation. Eitel et al. (2020) further demonstrated that offering learners proper guidance about cognitive and metacognitive strategies can improve their self-control of cognitive processing to reduce ECL and foster GCL. Additionally, Seufert (2018) argued that in different phases of self-regulation, learners need to invest cognitive and metacognitive resources in addition to dealing with the original learning task. The affordances of self-regulation impose cognitive load and might even cause cognitive overload (Seufert, 2018). Seufert (2018) analyzed the affordances of Zimmerman (2000) three phases of SRL in terms of ICL, ECL, and GCL. Meanwhile, external learning supports (e.g., prompts) have the potential to promote effective self-regulation processes, which can elicit the optimal amount of CL (Seufert, 2018). A handful of empirical studies (e.g., Liu and Sun, 2021; Sun and Liu, 2022) also illuminate how the employment of SRL-enabling tools for supporting SRL strategies can optimize cognitive load in AOCs.

In sum, researchers have established theoretical connections between SRL and CL and suggested how to optimize CL by externally supporting learners’ self-regulation. However, since this is an emerging research topic, limited studies have empirically investigated the underlying mechanisms through which temporally unfolding SRL processes have associations with ICL, ECL, and GCL. Additionally, to our knowledge, no studies have examined the relationship between SRL and CL in a specific course, especially in the context of AOL.

Student engagement (SE) refers to a student’s active participation and involvement in learning tasks and activities and consists of three different but related dimensions: behavioral, emotional, and cognitive (Fredricks et al., 2004). Behavioral engagement (BE) describes students’ observable behaviors while participating in academic activities that are crucial for attaining desired academic outcomes and preventing dropouts (Fredricks et al., 2004). This includes attention, concentration, effort, persistence, positive conduct, absence of disruptive behaviors, and involvement in curricular and extracurricular activities (Fredricks et al., 2004; Appleton et al., 2008). Emotional engagement (EE) describes students’ affective reactions (e.g., anger, anxiety, boredom, happiness, and interest) to teachers, peers, courses, and schools, their willingness to do the coursework, their sense of belonging in school, and their evaluation of school-related outcomes (Fredricks et al., 2004). Cognitive engagement (CE) describes thoughtfulness and willingness to exert effort to comprehend complex ideas and master difficult skills (Fredricks et al., 2004). It reflects students’ psychological investment in learning and strategic emphases on active self-regulation of skills and usage of deep learning strategies (Fredricks et al., 2004; Greene, 2015).

Prior research has adopted variable-centered approaches (e.g., correlation and regression) to associate SRL with SE in AOL (e.g., Pellas, 2014). For example, Sun and Rueda (2012) analyzed 203 college students’ self-reports of self-regulation and engagement after watching video recordings of lectures in a distance course. They found that self-regulation was significantly positively correlated with BE, EE, and CE, implying that students with higher levels of self-regulation demonstrated higher levels of engagement. The positive relationship between SRL and SE has been well established in variable-centered studies (Anthonysamy et al., 2020). Going beyond analyzing SRL behaviors from a variable-centered perspective, which assumes the same relations and average means for an entire population, recent studies (e.g., Pérez-Álvarez et al., 2020) increasingly concentrate on person-centered approaches to detect divergent SRL profiles and how those profiles differ regarding SE. These approaches are especially apt for studies conducted in AOL contexts where SRL behaviors vary greatly across individual students. For example, mapping SRL behavioral indicators with the clickstreams of 5,014 learners enrolled in an MOOC, Lan et al. (2019) employed K-means to find two types of learners (i.e., auditors and attentive) who shared similar patterns of SRL behaviors. They concluded that the attentive learners who followed the learning pathway intended by the instructors showed higher course engagement and completion rates than the auditors who accessed course content selectively and irregularly.

In sum, existing studies have illuminated the impacts of students’ SRL profiles on their engagement in AOCs, but most are limited to examining BE. Whether and how SRL profiles are associated with EE and CE remains unclear. Moreover, these studies distinguished SRL profiles according to frequency-based measures of SRL behaviors. To date, no studies have related divergent profiles of temporally unfolding SRL processes to the three types of SE.

The purpose of the current study is therefore threefold: (1) identifying student subgroups according to traces of online SRL behaviors; (2) examining the student subgroup differences in learning performance, CL, and SE; and (3) articulating and comparing behavior patterns of online SRL between the student subgroups. This study offers researchers both theoretical and methodological insights. Additionally, our research findings inform practitioners about how to design and deploy personalized SRL interventions in the context of AOL. Accordingly, the research questions are as follows:

**RQ1.** Can student subgroups be identified by the traces of SRL behaviors collected from the use of SRL-enabling tools to complete an AOC? If so, what are their characteristics?

**RQ2.** Do the identified student subgroups significantly differ in learning performance, cognitive load, and student engagement?

**RQ3.** How does this study differentiate the identified student subgroups according to their behavior patterns of online SRL?

## 2. Materials and methods

### 2.1. Participants and settings

We recruited 113 graduate students who had never attended research ethics courses before from universities in northern Taiwan. These participants were asked to complete an asynchronous online research ethics course. The course consisted of four learning units, each of which took participants approximately 40 min to complete. Twelve students were excluded because of data limitations, such as incomplete traces of SRL behaviors and insufficient learning time, leaving a final sample size of 101 students ($M_{age}$ = 24.21 years, $SD_{age}$ = 3.37, 53.5% female).

**Figure 1:**
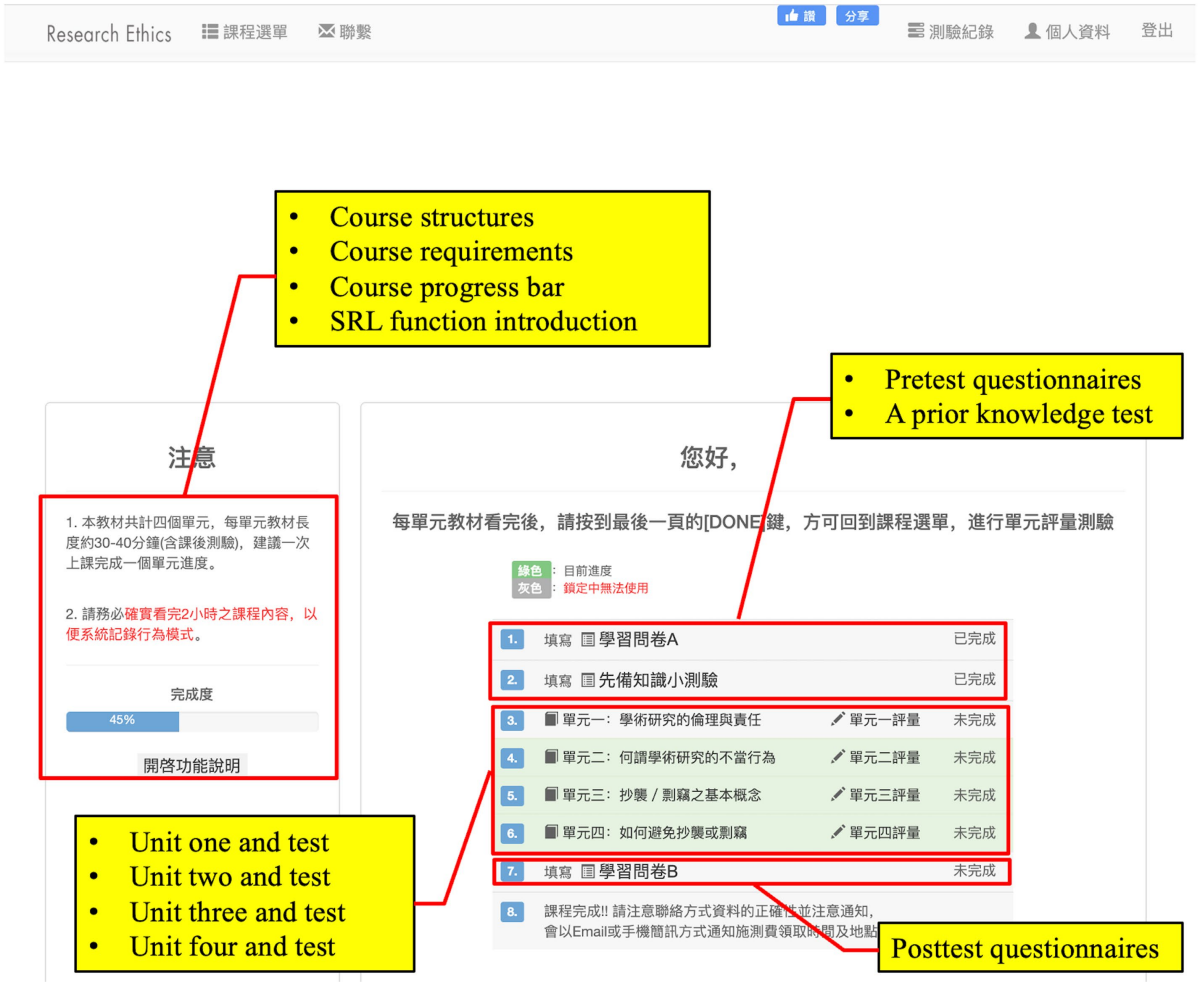
The user interface for unit selection and test taking.

**Figure 2:**
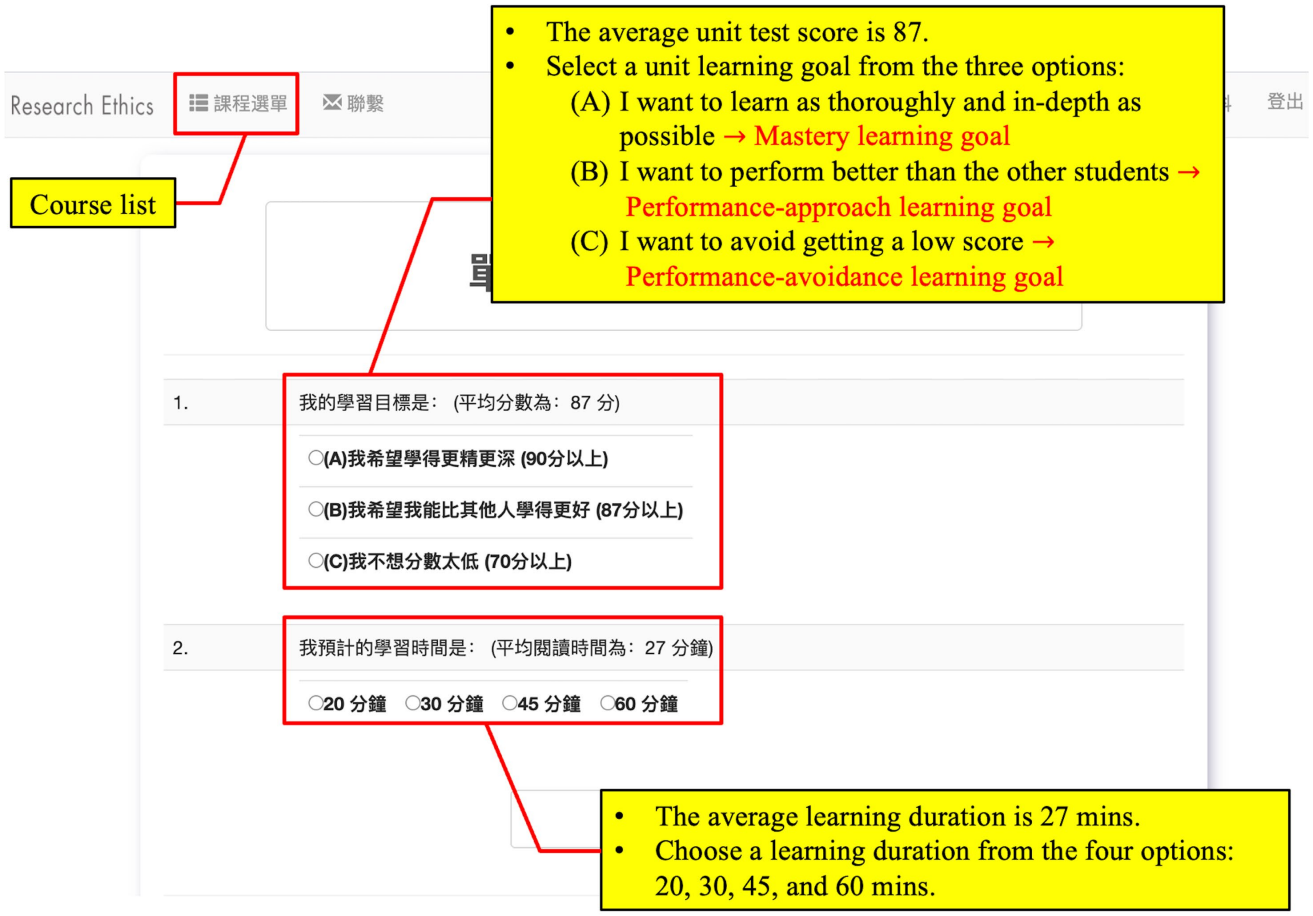
The user interface for goal and duration setting.

Sun and Liu (2022) designed the learning units according to Zimmerman (2000) three-phase SRL model and integrated Barnard et al. (2009) online SRL strategies in the form of tools into the three phases of SRL. In the forethought phase, learners selected a learning unit with reference to their personal interests and prior-knowledge test scores on the course list (Figure 1). Then, they were required to set a learning goal and a learning duration referring to previous learners’ averages on unit test scores and time-on-unit (Figure 2). Based on Elliot and Church (1997) achievement goal theory, we recommended that learners choose among three different learning goals: mastery, performance-approach, and performance-avoidance goals (Figure 2). According to the learning duration data collected by Sun et al. (2018, 2019), we provided four options: 20, 30, 45, and 60 min. If learners want to change the learning unit, they can click the “Course List,” which takes them back to the course list. From there, they can reselect a learning unit.

**Figure 3:**
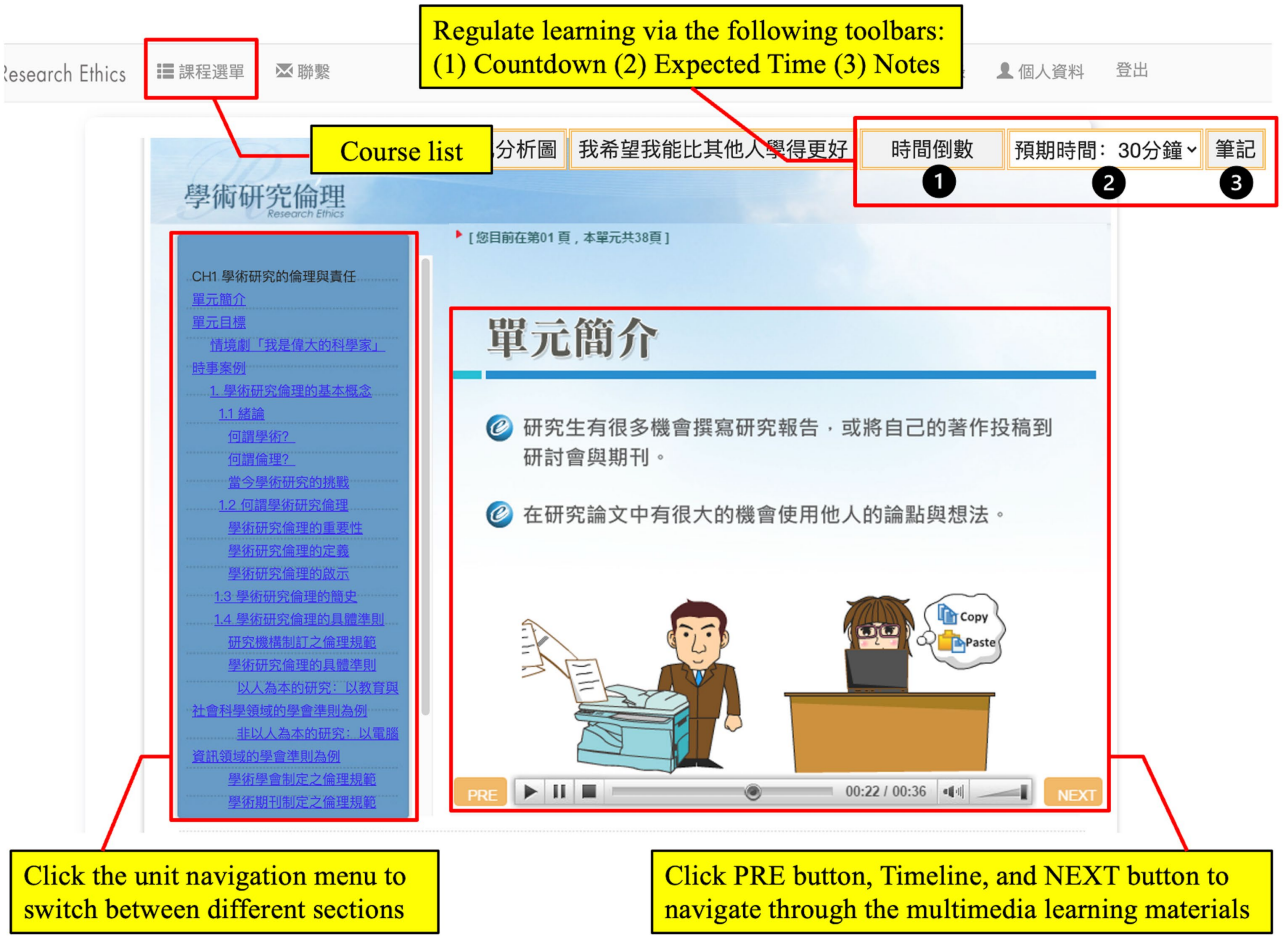
The user interface for “Countdown,” “Expected Time,” and “Notes.”

After plan making, learners proceeded to the performance phase in which they could implement SRL strategies *via* these tools to study multimedia learning materials (Figure 3). Students could watch and control learning materials with flash animation and switch between content sections freely by leveraging a navigation menu. Meanwhile, the top of the course interface displays a toolbar with three tools, namely, “Countdown,” “Expected Time,” and “Notes.” Learners can check how much time is left by clicking on “Countdown.” The information about the remaining time is masked in the absence of click actions for 5 s. When only 5 min are left, the “Countdown” icon will flash to remind learners to adjust their learning pace, such as resetting learning duration *via* “Expected Time.” When studying the materials, learners can use “Notes” to type in, delete, and save notes. While learning, if learners want to change the learning unit and learning goal, they can return to the course list by clicking the “Course List” to recreate their study plan.

**Figure 4:**
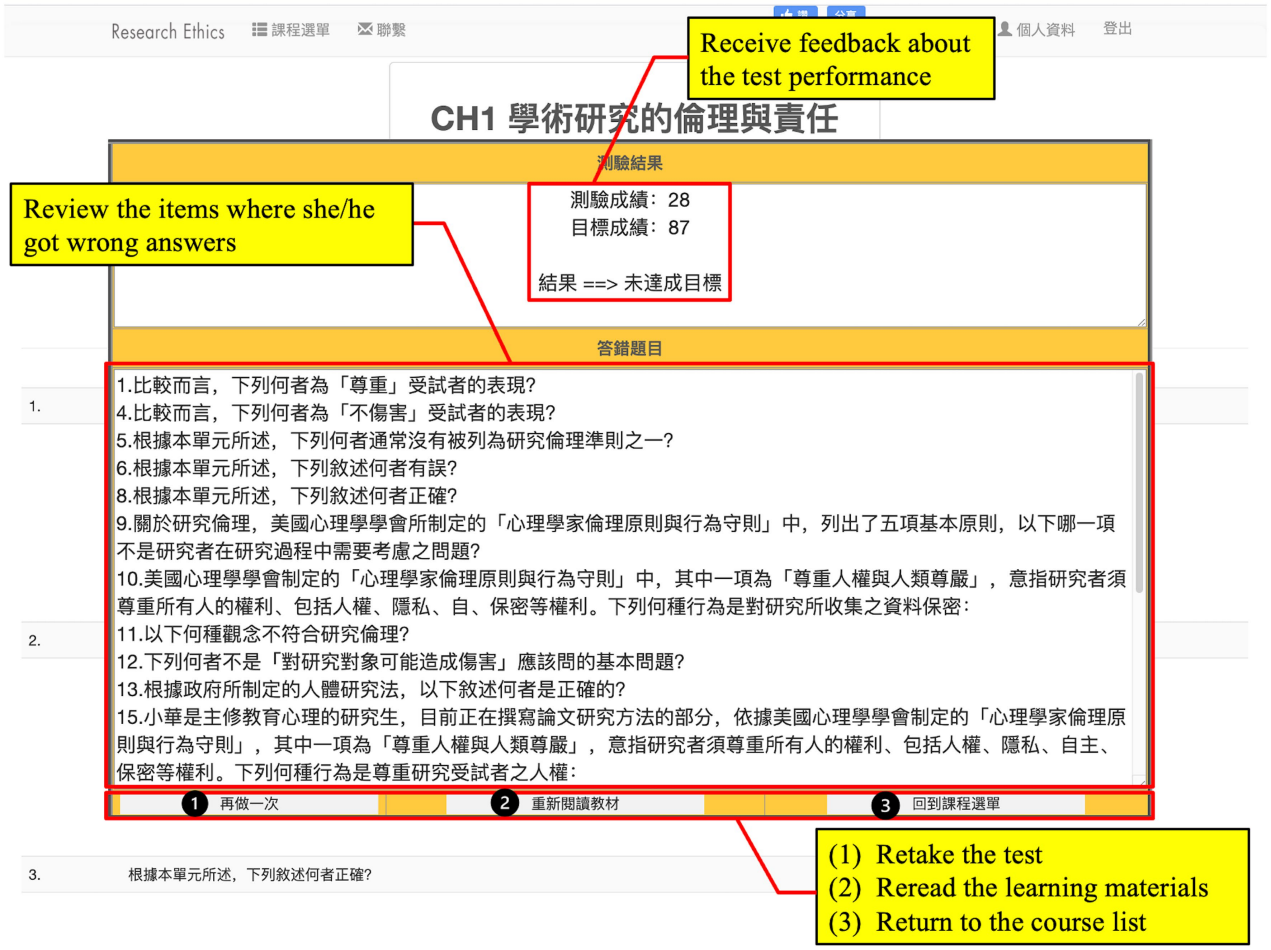
The user interface for test feedback.

After studying the learning materials, learners evaluate their performance by attending a unit test. Once finishing the test, learners received a performance feedback report including their test performance and the items they missed (Figure 4). Based on the feedback, learners determined whether to retake the unit test, review the learning materials, or start another learning unit. After finishing all the learning units, learners were asked to fill out cognitive load and student engagement questionnaires.

Considering the prevalence of digital multitasking and distraction in AOL settings, this study defines and identifies learners’ off-task behaviors in terms of Sun et al. (2018) study carried out in the same course. Specifically, off-task behaviors appear if there are 20 min of gap time between two consecutive keystrokes or clicks. It should be noted that we exclude the environmental structuring dimension since it is hard to measure based on action logs. Additionally, students were asked to pass the course independently. Thus, help-seeking strategies were not provided in the course. Nevertheless, when encountering technical problems, learners could contact instructors *via* email.

### 2.2. Data collection

We collected the participants’ SRL behaviors according to the coding scheme (Table 1) developed based on Zimmerman (2000) three-phase model and Barnard et al. (2009) online SRL strategies. This study developed 10 SRL behavior codes and embedded coding rules into the learning system. Once learners used the SRL-enabling tools or were off-task, the corresponding behaviors were detected and recorded automatically. For descriptions of each coded behavior, please see “Participants and settings.”

**Table 1:** The coding scheme of online SRL behaviors.

SRL phase	Online SRL strategy	Online SRL behavior	Code
Forethought	Goal setting	Selecting a learning unit	SU
		Choosing a mastery learning goal	G1
		Choosing a performance-approach learning goal	G2
		Choosing a performance-avoidance learning goal	G3
		Setting a learning duration	SD
Performance	Task strategies	Taking notes	TN
	Time management	Checking remaining learning time	CT
		Resetting a learning duration	RD
Reflection	Self-evaluation	Taking a unit test	TT
		Performing off-task behaviors	OT

Online unit tests were administered to evaluate the participants’ research ethics knowledge acquired in the course. Specifically, the four tests contained 25, 13, 17, and 16 multiple-choice items, and the maximum score of each test was 100 points. We averaged the four test scores for each participant as his or her learning performance score. All the items were developed and applied by Sun et al. (2018, 2019).

The cognitive load questionnaire by Leppink et al. (2013) was adapted to measure the participants’ ICL (three items), ECL (three items), and GCL (four items). All the items were assessed on an 11-point Likert scale (0 = *strongly disagree*, 10 = *strongly agree*). The Cronbach’s α was.92, 0.90, and.92 for ICL, ECL, and GCL, respectively.

The student engagement questionnaire by Fredricks et al. (2005) was adapted to measure the participants’ BE (five items), EE (six items), and CE (eight items). All the items were assessed on a 5-point Likert scale (1 = *strongly disagree*; 5 = *strongly agree*). The Cronbach’s α was.71, 0.92, and.87 for BE, EE, and CE, respectively.

### 2.3. Data analysis

A sequence analysis with the R package *TraMineR* (Gabadinho et al., 2011) was undertaken to visualize and compare the sequences of behaviors captured based on our coding scheme. The first step of implementing between-sequence comparisons was obtaining edit distances for pairs of sequences as the minimal cost, in terms of inserting, deleting, and substituting sequence behaviors to transform one sequence into another. Specifically, a dissimilarity matrix was established using the optimal matching algorithm with an insertion/deletion cost of 1 and a substitution cost matrix based on observed transition rates between behaviors. Based on the dissimilarity matrix, we employed K-medoids with the R package *fpc* (Henning, 2020) to organize these behavior sequences into homogeneous clusters. Meanwhile, the average silhouette method was used *via* the R package *factoextra* (Kassambara and Mundt, 2020) to find the optimal number of clusters. To label the identified clusters, we used *TraMineR* to plot the behavior distribution and representative sequences for each cluster. Additionally, Welch’s independent *t* tests were performed to quantify the differences between the clusters regarding learning performance, cognitive load, and student engagement.

This study ran an LSA (Bakeman and Quera, 2011) using GSEQ 5.1 software to identify, visualize, and compare significant transition patterns among the SRL behavior codes demonstrated by the clusters. First, the SRL behaviors were coded into two-behavior sequences according to the chronological order. Second, to tally transitions among these behavior codes, the LSA produced a transitional frequency matrix in which each cell represents the number of times that one particular “given” code transitions immediately to another “target” code. Third, after generating the transitional frequency matrix, it proceeded to compute a transitional probability matrix. Specifically, a transitional probability represents the ratio of the frequency of a cell to the frequency for that row. Fourth, it computed an adjusted residual (i.e., *z* score) for each transition to determine whether the transitional probability showed significant deviation from its expected value. A *z* score above 1.96 implies that the transition from one code to another successor code reaches statistical significance (*p* < 0.05). Last, the behavioral transition diagram for each cluster was created according to the significant transition sequences.

An ENA (Shaffer, 2017) was implemented *via* the ENA Web Tool (version 1.7.0; Marquart et al., 2018) to model, visualize, and compare the cooccurrences of the codes for the two groups. First, this study defined the SRL behavior codes as the ENA codes, the participants as the units of analysis, and two consecutive SRL behaviors as the moving stanza. Second, based on the temporal behaviors, it created an adjacency matrix per stanza per participant, summed the adjacency matrices across all stanzas into a cumulative adjacency matrix for each participant, and then converted each resulting cumulative adjacency matrix into a normalized adjacency vector in a high-dimensional space. Third, it constructed a projected ENA space by performing dimensional reduction on the vectors *via* means rotation (MR) and/or singular value decomposition (SVD). MR is performed to position group means along a common axis to obtain the largest differences between the groups, whereas SVD is utilized to generate orthogonal dimensions that represent the most variance explained by each dimension. Fourth, it produced each participant’s epistemic network graphs in this space employing two coordinated representations: (1) a projected point graph, which showed the location of his or her network in the two-dimensional ENA space, and (2) a weighted network graph where nodes represent the codes and edges correspond to the relative frequency of links between any pair of nodes. The node positions are fixed across all networks and determined through an optimization routine minimizing the distance between the projected points and the centroids of their corresponding network graphs. Last, to compare the network graphs between the groups, we created ENA subtraction graphs by subtracting the weight of each connection in one group network from the corresponding connections in the other. In addition, the distributions of the projected points for the groups were compared using two-sample *t* tests.

## 3. Results

### 3.1. RQ1: Identifying student subgroups based on the SRL behavior sequences

**Figure 5:**
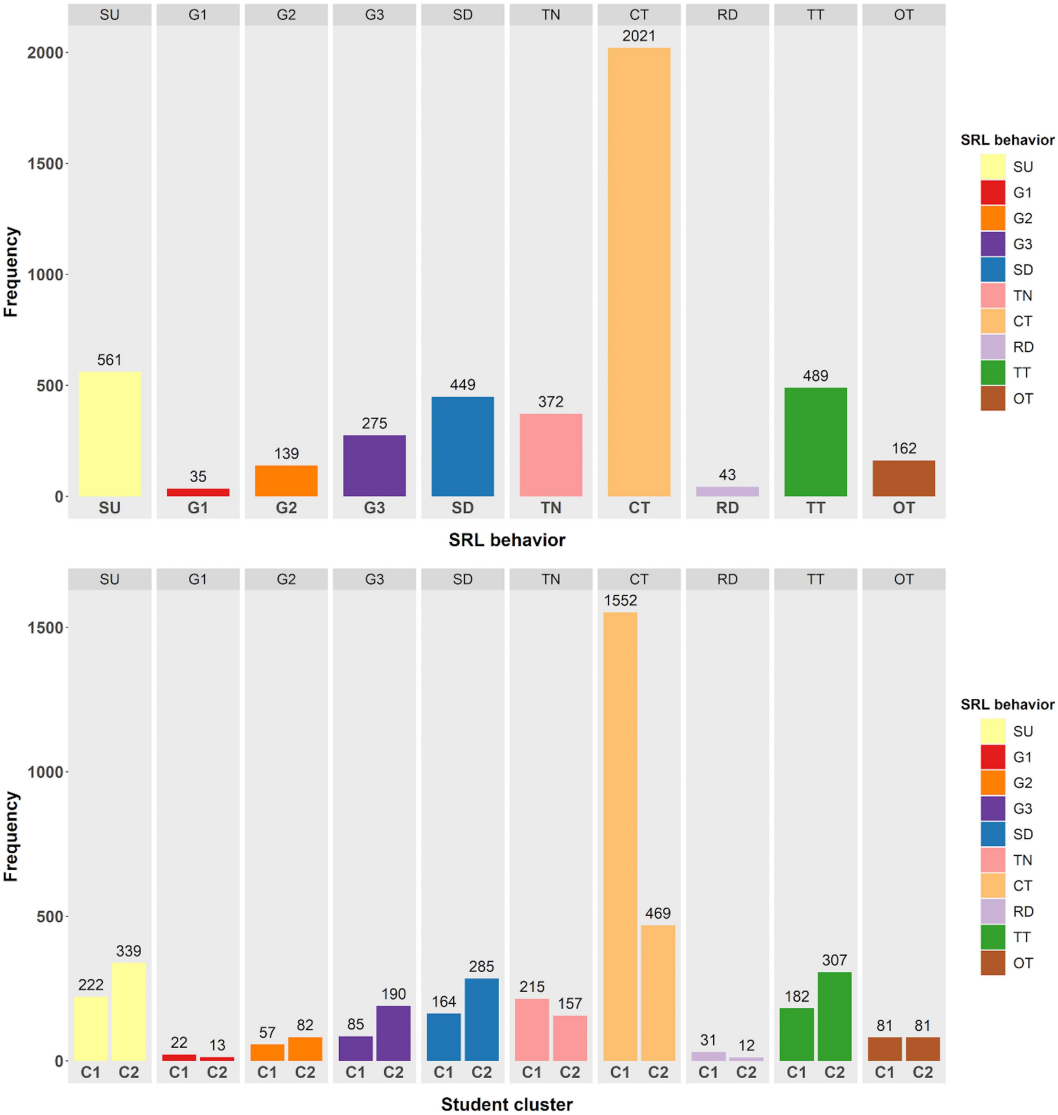
The frequencies of SRL behaviors for the overall sample (top) and the two clusters (bottom). C1 = Cluster 1; C2 = Cluster 2.

**Figure 6:**
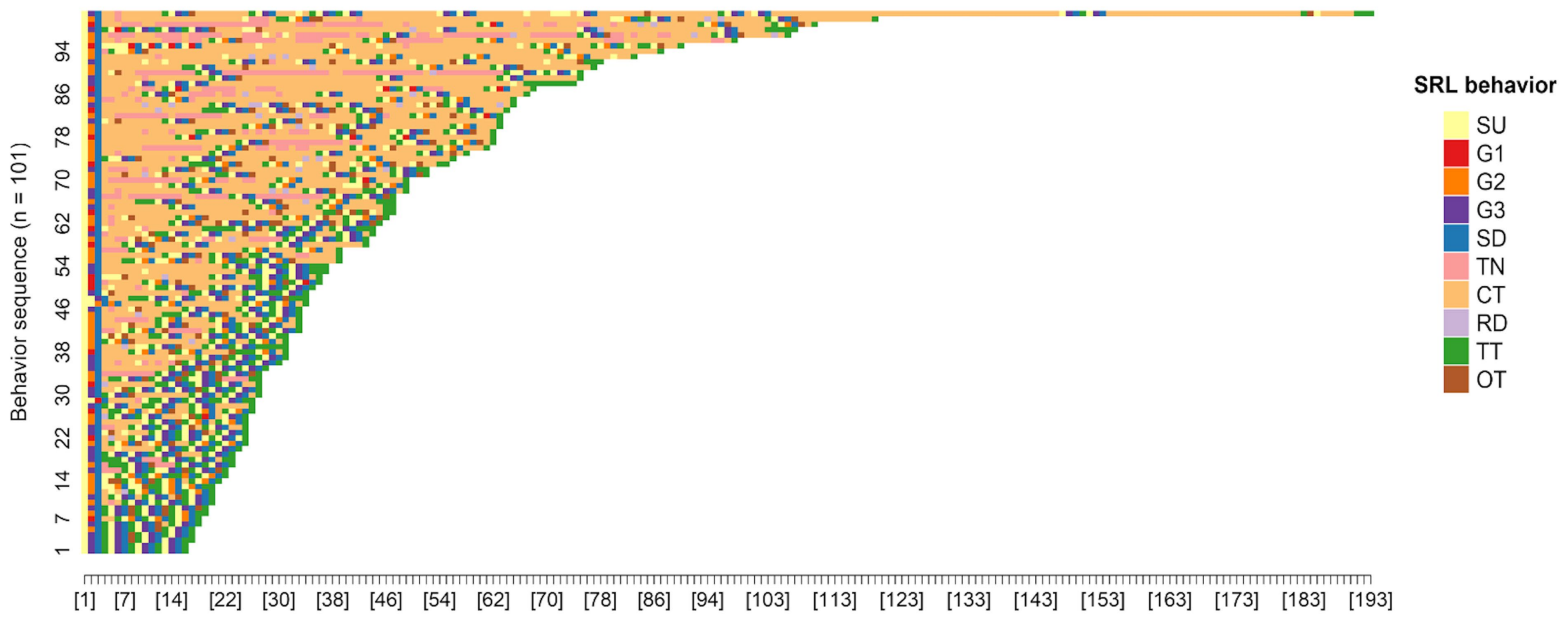
Plot of SRL behavior sequences for the whole sample.

We collected a total of 4,546 SRL behaviors generated by the whole sample. Figure 5 displays the behavior frequencies. Moreover, this study visualized behavior sequences for each participant in Figure 6. Each point on the x-axis of the figure represents a corresponding position of a behavior sequence, and each value on the y-axis represents a single participant. Each line shows a series of SRL behaviors, as distinguished by different colors, that an individual learner executed during the course. Figure 6 reveals that for the learning of each unit, almost all participants start with selecting a learning unit, then setting a learning goal and duration, and end up taking a unit test. It also shows that the vast majority exhibited unique and personalized SRL behavior sequences, especially in the forethought and performance phases. For example, some students are more inclined to set performance-avoidance goals. Moreover, the sequence length widely varies from 16 to 193, indicating that some students performed longer sequences of behaviors. Such differences suggest that learner heterogeneity in temporal SRL behaviors may exist.

**Figure 7:**
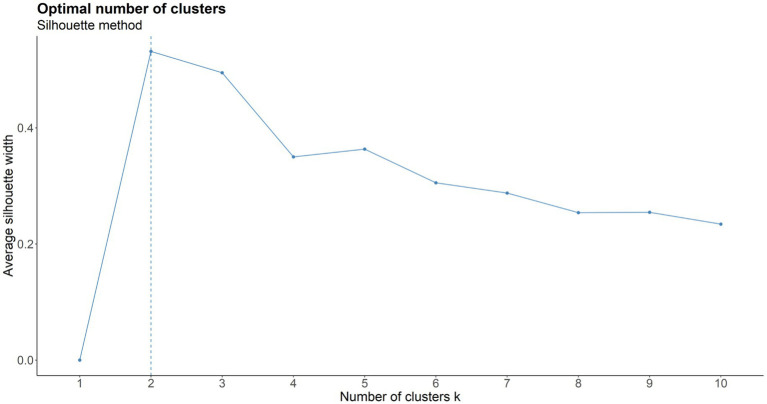
Average silhouette method for the selection of optimal clusters.

**Figure 8:**
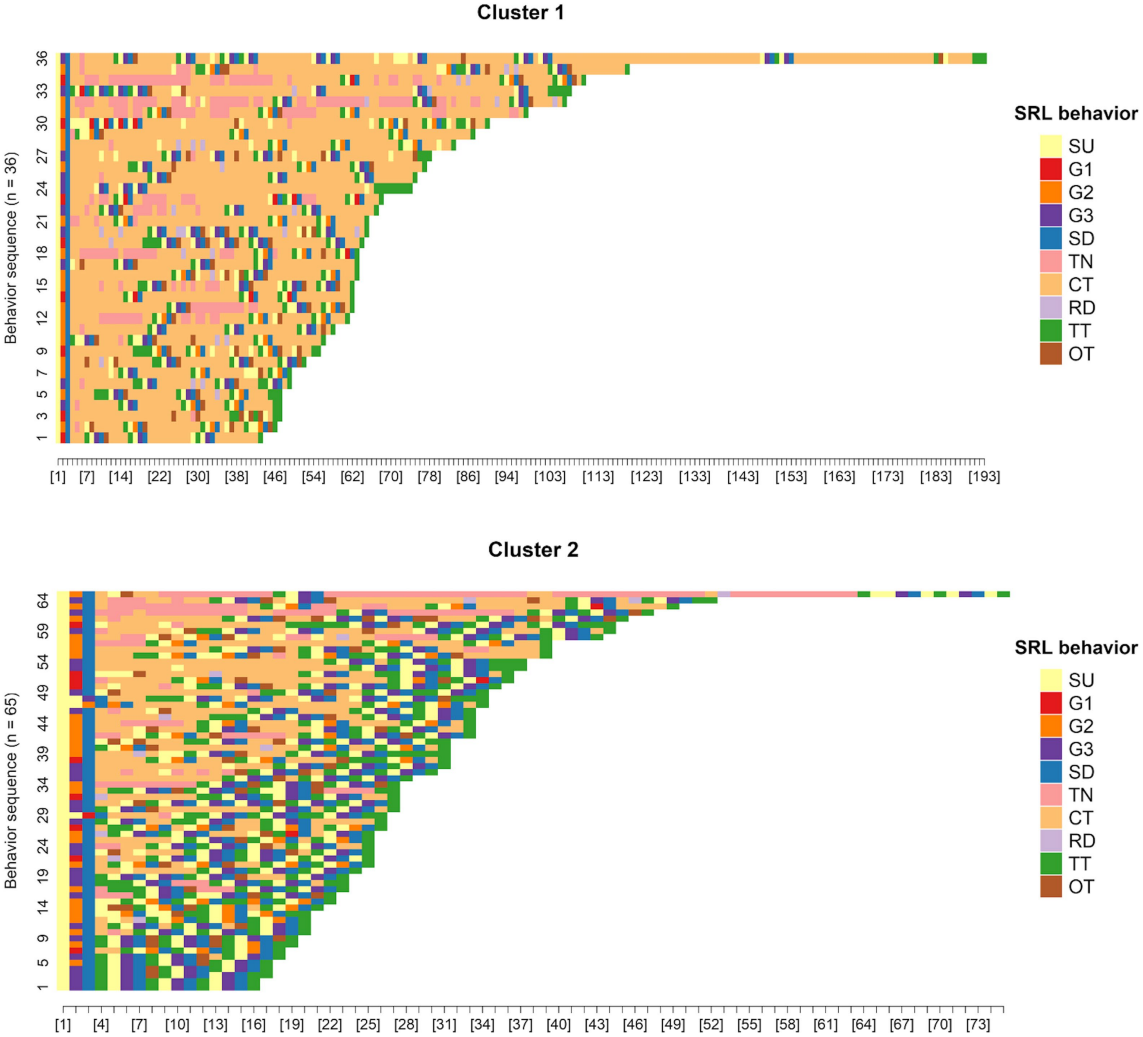
Plots of SRL behavior sequences by clusters.

**Figure 9:**
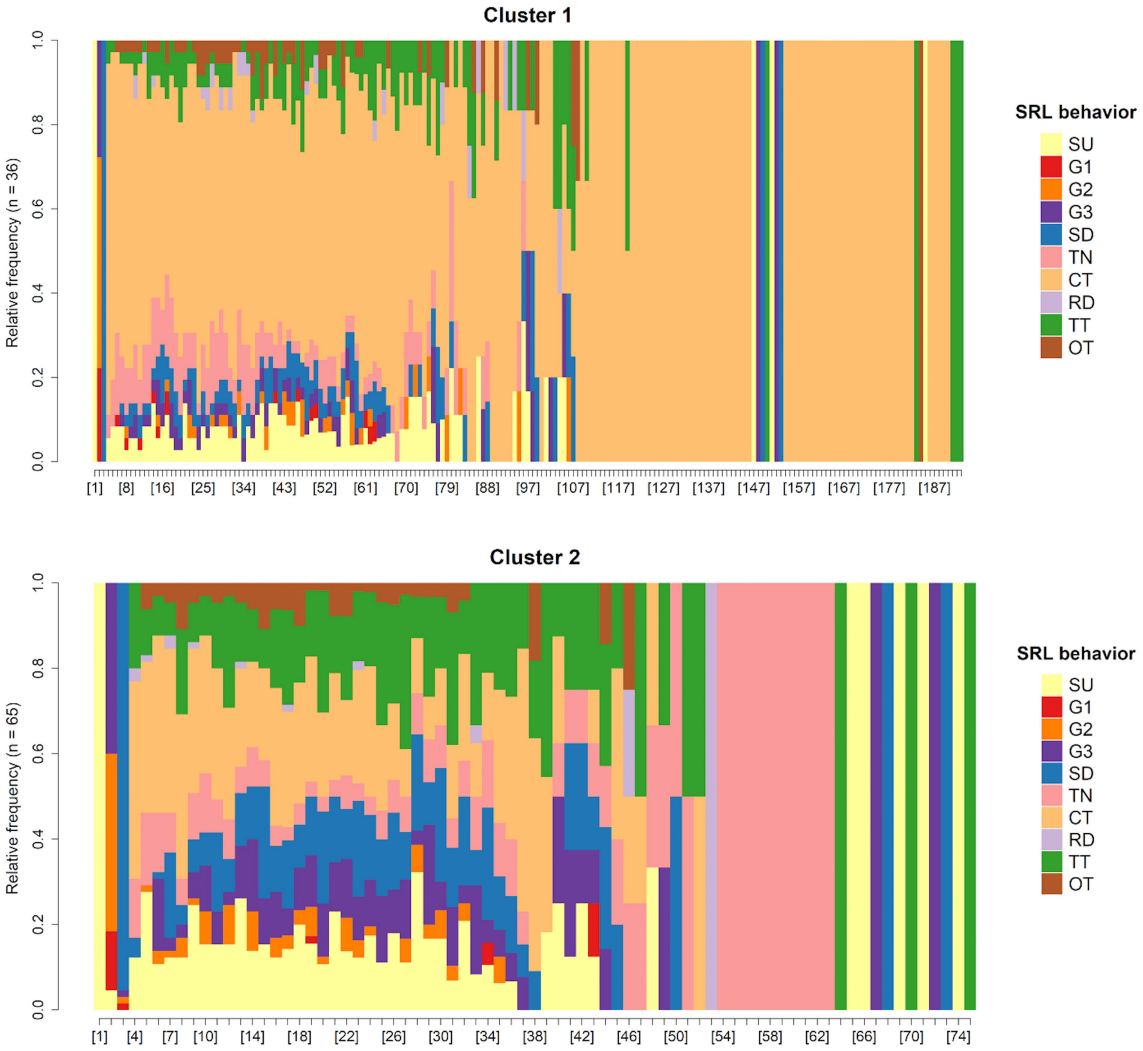
SRL behavior distribution plots by clusters.

**Figure 10:**
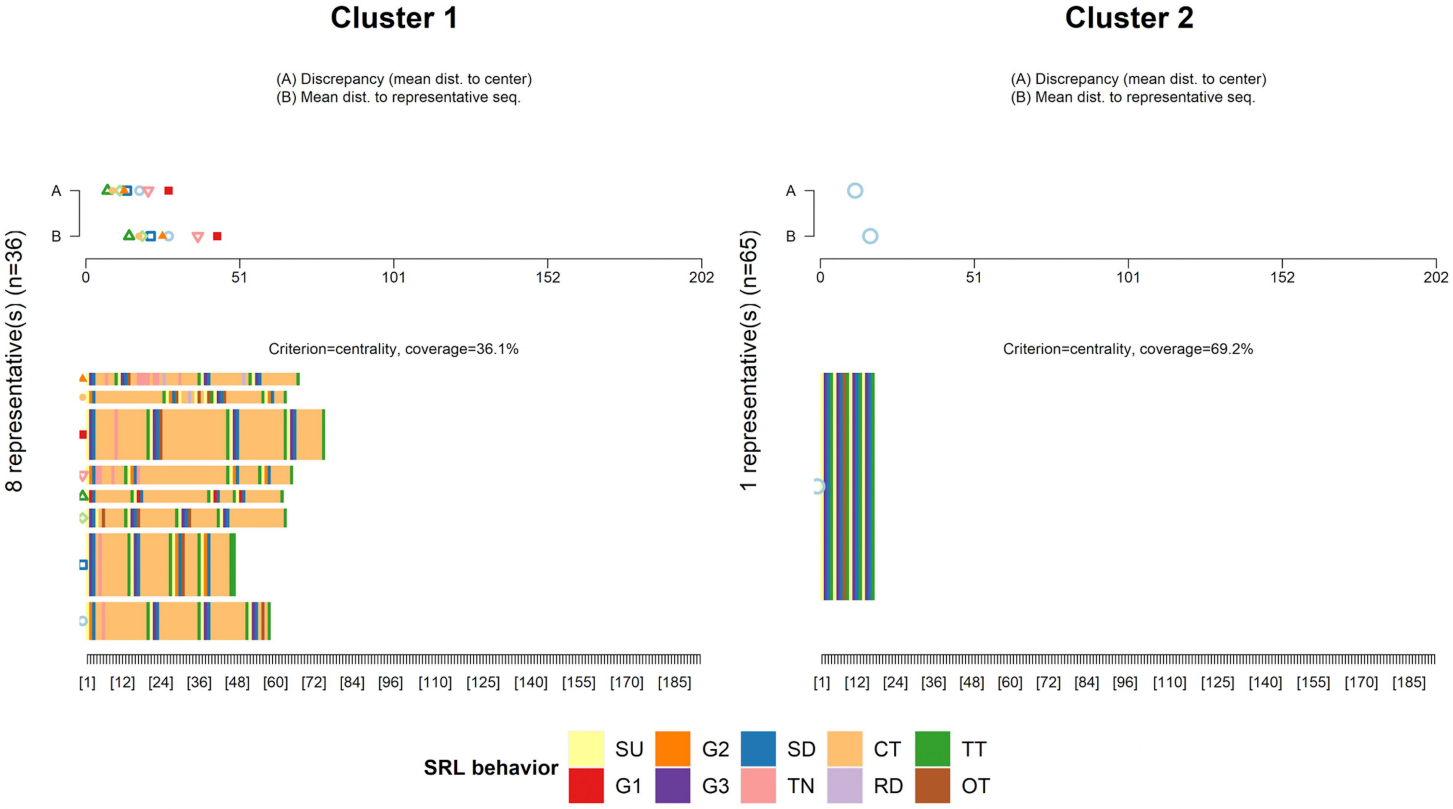
Representative sequence plots by clusters.

According to Figure 7, *K* = 2 was chosen as the ideal number of clusters. Subsequently, the partitioning around medoids (PAM) algorithm was used on the dissimilarity matrix obtained from sequence analysis, classifying participants into two clusters: Cluster 1 (*n* = 36) and Cluster 2 (*n* = 65). Figures 8–10 illustrate that between-cluster heterogeneity and within-cluster homogeneity became readily apparent in the two-cluster SRL behaviors. Although Cluster 1 had a smaller number of participants than Cluster 2, the former exhibited more frequent behaviors and longer sequence lengths (Figure 8). Both clusters’ state distributions of SRL behaviors from the beginning to the end of the course are depicted in Figure 9. Figures 5, 9 show that students from Cluster 1 devoted more effort to the performance phase, especially in time management, whereas those from Cluster 2 focused more on the regulatory activities of the forethought and reflection phases. Moreover, learners from Cluster 2 preferred setting performance-avoidance learning goals.

To further explore the differences in how learners from different clusters regulated their learning, we extracted the medoid, or most central sequences, from the two clusters as their representative sequences (Figure 10). Cluster 1 was represented by eight representative sequences, which were long and covered 36.1% of the sequences. In Cluster 2, we identified only one representative sequence, which was relatively short in length but gave 69.2% coverage. The sequences mined from Cluster 1 showed that the learners adaptively went through the three phases of SRL and demonstrated sophisticated behavior transitions. For example, when facing different learning units, learners modified learning goals by self-evaluating their performance at that time. When studying unit materials, they executed strategies of notetaking and time management depending on their learning needs. After off-task behaviors occurred, the learners usually checked the remaining learning time to adjust the subsequent learning pace. In contrast, the representative sequence identified in Cluster 2 indicated that although three-phase SRL was triggered, the participants predictably repeated the same set of SRL behaviors without any modification of strategies across the four learning units. Interestingly, they oriented themselves toward performance-avoidance learning goals. Given the findings above, we labeled Cluster 1 and Cluster 2 as the high online self-regulated learning group (H-SRL) and the low online self-regulated learning group (L-SRL), respectively.

### 3.2. RQ2: Comparing the subgroups’ learning performance, cognitive load, and student engagement

Table 2 shows that the H-SRL (*M* = 87.31, *SD* = 5.43) had significantly better learning performance than the L-SRL (*M* = 83.49, *SD* = 10.32). Moreover, the H-SRL (*M* = 7.42, *SD* = 5.59) exhibited significantly lower ECL than the L-SRL (*M* = 10.88, *SD* = 7.15). The H-SRL (*M* = 33.11, *SD* = 5.26) experienced significantly greater GCL than the L-SRL (*M* = 30.26, *SD* = 5.35). However, the *t* test results on ICL revealed nonsignificant differences between the groups. For the SE, the CE of the H-SRL (*M* = 29.28, *SD* = 4.37) was significantly higher than that of the L-SRL (*M* = 26.83, *SD* = 5.16), but no significant differences were found in BE and EE. According to Cohen (1988), the effect size was small for learning performance and medium for ECL, GCL, and CE.

**Table 2:** The results of Welch’s independent *t* tests on learning performance, cognitive load, and student engagement between the two groups.

Variables	H-SRL (*n* = 36)		L-SRL (*n* = 65)		*t* (*df*)	*p*	Cohen’s *d*
	*M*	*SD*	*M*	*SD*			
Learning performance	87.31	5.43	83.49	10.32	2.43 (98.82)*	0.017	0.46
Intrinsic cognitive load	13.22	5.91	14.74	7.64	−1.11 (88.35)	0.271	−0.22
Extrinsic cognitive load	7.42	5.59	10.88	7.15	−2.69 (87.72)**	0.009	−0.54
Germane cognitive load	33.11	5.26	30.26	5.35	2.59 (73.46)*	0.012	0.54
Behavioral engagement	19.39	3.04	18.46	2.72	1.52 (65.96)	0.132	0.32
Emotional engagement	19.83	4.75	18.09	4.73	1.77 (72.17)	0.081	0.37
Cognitive engagement	29.28	4.37	26.83	5.16	2.52 (82.97)*	0.013	0.51

*p < 0.05, **p< 0.01, ***p < 0.001

### 3.3. RQ3: Examining the subgroups’ behavior patterns of SRL

**Figure 11:**
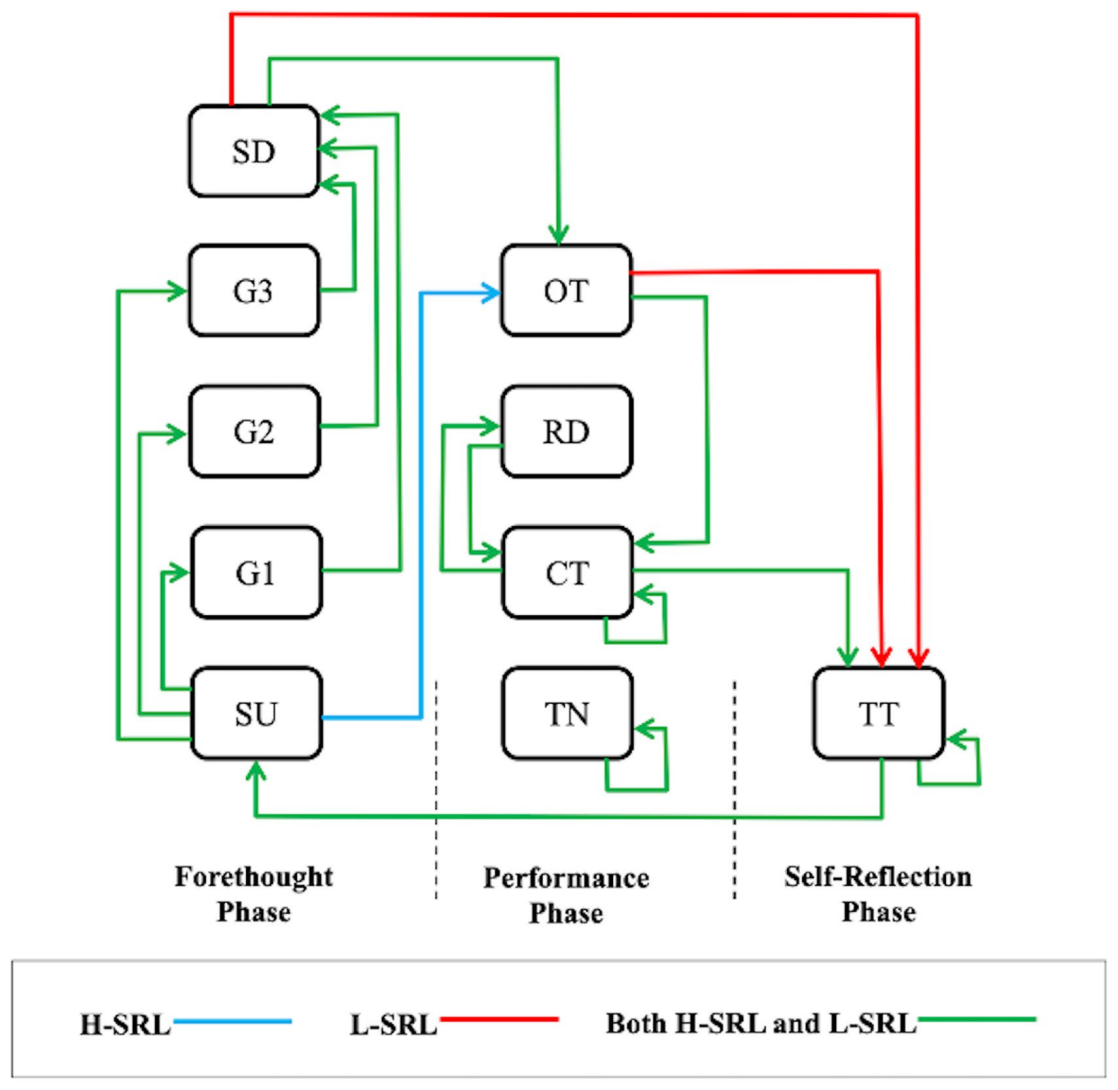
Behavior patterns of the H-SRL and L-SRL.

Supplementary Appendix A presents the LSA results. The significant behavior patterns are portrayed in Figure 11, where the behavior codes are signified with round rectangles and the significant transitions are signified with arrows. Both groups shared some common transition sequences. In the forethought phase, the participants started by choosing a learning unit, then settled on a learning goal, and ended up with setting a learning duration (SU→G1, G1→SD, SU→G2, G2→SD, SU→G3, and G3→SD), indicating that they usually acted in compliance with the tools supporting goal setting. In the performance phase, they repeatedly took notes (TN⇄TN) and usually performed time management-related behavior transitions such as repeatedly checking remaining learning time (CT⇄CT), checking remaining learning time before attempting a test (CT→TT) and switching between checking remaining time and resetting learning durations (CT⇄RD). These sequences illustrate that students are required to invest much effort in organization and time management in AOL contexts. Additionally, it should be noted that both groups exhibited off-task behaviors after setting a learning duration (SD→OT) or before checking remaining learning time (OT→CT). This kind of behavior transition indicates that off-task behaviors are difficult to prevent in AOL environments, but SRL-enabling tools can offer remedy support, such as displaying the remaining learning time. In the self-reflection phase, after completing a unit test and receiving system feedback, the learners either attended the same unit test again (TT⇄TT) or started another learning unit (TT→SU), indicating that learners evaluated their learning according to the unit test and system feedback and then made learning adjustments. However, some different behavioral transfers were found between the two groups. The H-SRL usually went off-task after selecting a learning unit (SU→OT), indicating that learners disengaged from the forethought phase, possibly because they struggled to determine an appropriate learning goal and learning duration by themselves. In contrast, the L-SRL directly attempted a test after setting a learning duration (SD→TT) or undertaking off-task activities (OT→TT), indicating that the L-SRL gravitated more toward unit tests to pass exams through minimal engagement.

The results of ENA showed that the x-axis corresponding to MR explained 23.4% of the variance in the network, while the y-axis corresponding to SVD explained 28.9% of the variance in the network. Moreover, two-sample *t* tests were applied to examine whether the network centroids (colored squares surrounded by dashed-border rectangles representing 95% confidence intervals) for the two groups differed along both the x-axis and the y-axis. We found a significant difference between the H-SRL (*M* = −1.34, *SD* = 0.93) and the L-SRL (*M* = 0.74, *SD* = 1.16) on the x-axis (*t* = −9.86, *df* = 86.81, *p* < 0.001) but a nonsignificant difference between the H-SRL (*M* = 0.00, *SD* = 1.34) and the L-SRL (*M* = 0.00, *SD* = 1.79) on the y-axis (*t* = 0.00, *df* = 90.26, *p* = 1.00). These findings indicate that the H-SRL made stronger connections to G1, TN, CT, and RD, whereas the L-SRL made stronger connections to G3 and TT.

**Figure 12:**
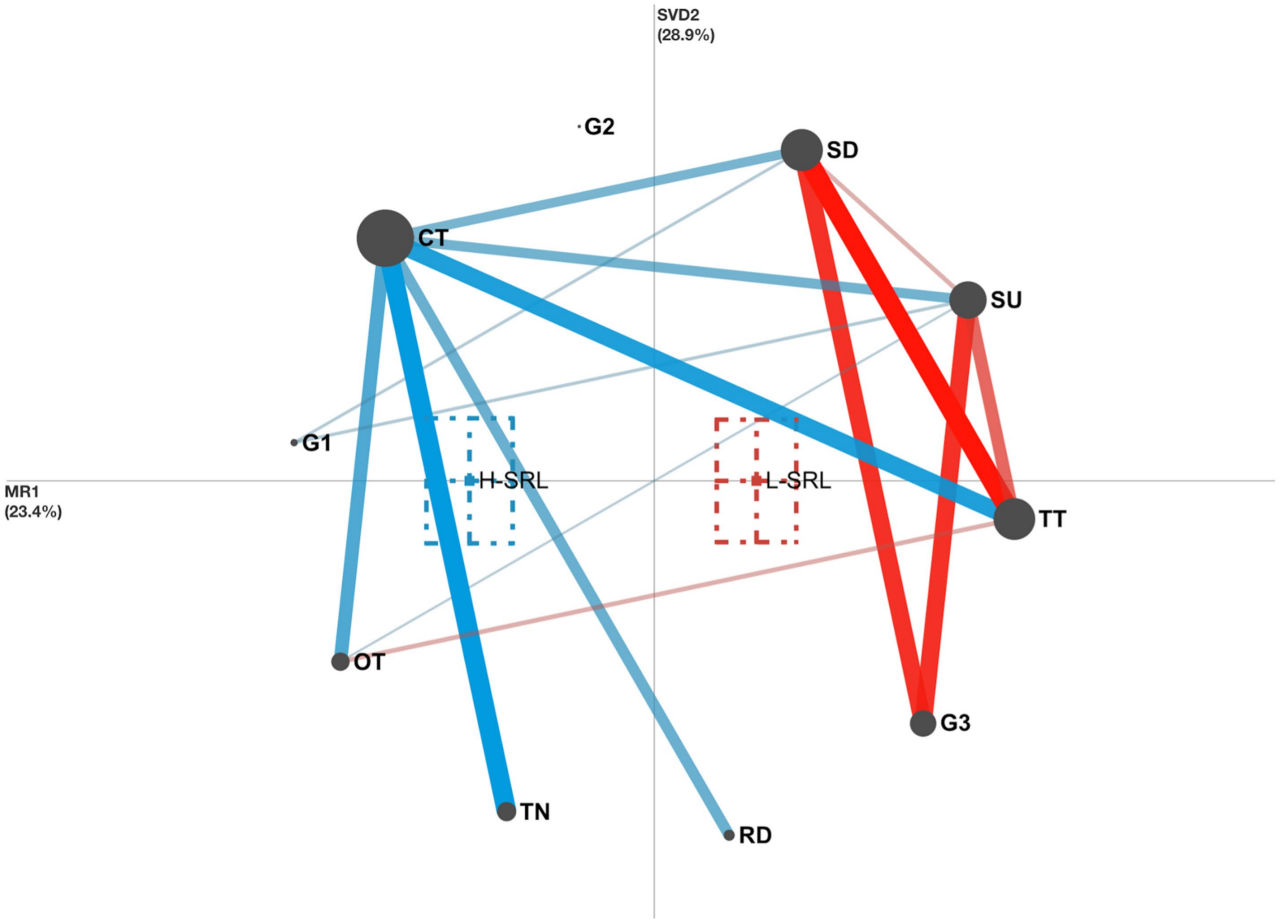
Comparison between the H-SRL (blue) and L-SRL (red) groups. Blue edges represent stronger associations in the H-SRL network; red edges represent stronger associations in the L-SRL network.

The ENA subtraction graph (Figure 12) was used to compare the mean networks of these two groups. Specifically, the H-SRL displayed stronger connections of SU and SD with G1 and weaker connections of SU and SD with G3 than the L-SRL, indicating that the H-SRL tended to choose mastery learning goals, while the L-SRL tended to set performance-avoidance learning goals. Moreover, the H-SRL showed more associations related to TN, CT, and RD and fewer associations related to TT than the L-SRL, indicating that the H-SRL preferred enacting organization and time management strategies to master the course content, while the L-SRL focused more on the unit tests than on the course materials. The H-SRL exhibited stronger links between OT and SU and CT and weaker links between OT and TT than the L-SRL. These links indicate that the H-SRL was more likely to exhibit off-task behaviors while planning for the learning units and usually checked the remaining learning time when off-task behaviors occurred. In contrast, when continuing learning was impeded due to off-task activities, the L-SRL was more inclined to start taking the unit tests rather than shifting back to reading the unit materials.

## 4. Discussion

This study found heterogeneity in students’ behavioral processes for online SRL. Specifically, we classified the participants into two clusters (i.e., H-SRL and L-SRL) according to their traces of SRL behaviors derived from an AOC with SRL-enabling tools. We found that the H-SRL obtained higher learning performance than the L-SRL. This finding is partially consistent with Cicchinelli et al. (2018), who leveraged behavioral trajectories of SRL codified from trace data derived from an LMS to detect student subgroups. They found the highest test scores in the group who performed SRL behaviors in regular and structured ways rather than in others who rarely or irregularly engaged in SRL activities.

Additionally, the H-SRL experienced lower ECL and higher GCL than the L-SRL, which verifies the theoretical assumption that learners’ self-regulation of learning processes has associations with their cognitive loads (Seufert, 2018). Moreover, these findings support the view that how learners process instruction relates to ECL and depends on learners’ abilities and willingness to exert self-control (Eitel et al., 2020). In this study, compared to the L-SRL, the H-SRL who exerted more self-control of their cognitive processing (e.g., checking learning time frequently) showed lower ECL. Additionally, the findings substantiate another assertion that the use of learning strategies and external learning supports is associated with GCL (Klepsch and Seufert, 2020). In this study, the H-SRL who engaged more with SRL strategies *via* the SRL tools, particularly time management and notetaking, experienced higher GCL than the L-SRL. Another possible reason for this result is that in contrast with the L-SRL, the H-SRL bore lower ECL, freeing up more mental resources for germane processes to maximize learning. Additionally, the H-SRL showed more CE than the L-SRL, which aligns with the findings of Kim et al. (2021), who demonstrated the relationship between SRL strategy use and CE in AOCs. They found that students who more frequently performed resource management strategies (e.g., time management) showed higher CE.

The above findings suggest that although the SRL-enabling tools were provided to support SRL strategies in AOL environments, not every learner will take advantage or glean the benefits of such tools to regulate their learning processes well. It is highly possible that some learners ignore or do not comply with the provided SRL support (Bannert et al., 2015). Due to poor compliance with support, learners’ regulation was not well aligned with the learning processes, or they failed to engage in deeper learning processes (Seufert, 2018).

The LSA results showed that both groups went through three-phase SRL cycles and executed many identical behavior transitions among SRL behaviors, which implies that the SRL-enabling tools, to some extent, can facilitate students’ implementation of SRL strategies in AOL. However, we also noticed that both groups performed off-task behaviors in the performance phase. This is not surprising, as opportunity costs for studying are relatively high when students are in AOCs (Eitel et al., 2020). Opportunity costs reflect events or activities that one must delay or sacrifice to achieve an academic goal (Wolters and Brady, 2021). For example, it is challenging to persist in engaging with an AOC when the mobile phone is in reach or when friends are present. To address this issue, as suggested by Kim et al. (2021), educators should design AOCs in a way that is helpful to sustain students’ engagement throughout the course. Additionally, the LSA results also reveal the differences in behavior transitions between the groups. For the H-SRL, off-task situations were detected in the forethought phase. One possible explanation is that insufficient reference information provided in the forethought phase made learners struggle to accurately judge the difficulty of course content and thus hesitate to set learning goals and learning durations. This explanation is underpinned by Hwang et al. (2021), who report that students who referred to peers’ suggestions for self-regulation were more likely to set appropriate learning goals in an AOC with SRL support. In contrast, the L-SRL displayed more transition sequences related to taking tests. Specifically, when encountering some challenges, such as distraction or managing learning processes independently, the L-SRL tended to avoid such challenges by attempting unit tests directly, suggesting that the L-SRL had a strong tendency to follow surface learning approaches. Surface learning approaches are characterized by weak learner commitment toward studying, low engagement with learning content, and high concentration on assessment and are negatively associated with learning performance (Matcha et al., 2019; Taub et al., 2022). Similarly, many prior studies on SRL also demonstrated the adoption of surface learning approaches in AOL (Loeffler et al., 2019). For example, based on the use of study tactics extracted from trace data that an LMS captured, Saint et al. (2020b) identified four learner strategy groups (i.e., active agile, summative gamblers, active cohesive, and semiengaged groups) and reported that the summative gamblers group tended to use surface learning approaches and underperformed on course exams compared with other groups. Specifically, this group mostly focused on summative assessments and exhibited suboptimal learning behaviors such as jumping straight to a summative test after goal setting.

We conducted an ENA to confirm and complement the LSA findings. Unlike the LSA, which generated directional transition sequences, the ENA quantitatively compared the two groups’ networks of co-occurrences between behaviors and uncovered the group differences in specific network connections in more detail. Specifically, a significant difference was found in the co-occurrence networks between the two groups. Moreover, the ENA subtraction graph showed that the H-SRL had stronger associations between selecting learning units and performing off-task behaviors, whereas the L-SRL had stronger associations between taking unit tests with setting learning durations and performing off-task behaviors, which confirmed the LSA findings. More interestingly, in contrast to the LSA results that both groups shared some common behavior patterns, the ENA subtraction graph unveiled the group differences in these behavior patterns. Specifically, the H-SRL made more connections to setting mastery learning goals and managing learning time, which echoes prior review research by Wolters and Brady (2021), who reported positive correlations between college students’ use of time management strategies and the adoption of mastery learning goals. In contrast, the L-SRL made more connections to setting performance-avoidance learning goals and taking unit tests, which verified the LSA finding that the L-SRL preferred surface learning approaches. Similar findings were also reported by Jovanović et al. (2017), who mined five student groups (i.e., intensive, strategic, highly strategic, selective, and highly selective groups) according to students’ learning sequences representing their interactions with an LMS and revealed that the intensive and strategic groups outperformed the highly selective group in exam performance. They found that the intensive and strategic groups displayed mastery-goal orientation and actively practiced different learning strategies to adapt to the course requirements, whereas the highly selective group exhibited performance-goal orientation and typically employed surface learning approaches.

## 5. Conclusion

The present study contributes to research in the field of SRL in several ways. First, we examined the temporal dynamics of students’ SRL behaviors in the context of AOL by identifying and visualizing potential student subgroups (i.e., H-SRL and L-SRL) based on students’ trajectories of online SRL behaviors. Second, we investigated whether and how the differences in SRL behavioral trajectories are associated with AOL success by (1) testing the student subgroups for differences regarding learning performance, cognitive load, and student engagement and (2) uncovering the SRL behavior patterns of the subgroups. Third, this study provided empirical evidence for the association of the self-regulation of learning processes with cognitive load and student engagement. We found that the H-SRL had lower ECL and higher GCL and CE than the L-SRL. Last, this study is the first attempt to combine LSA and ENA to articulate and compare behavior patterns of SRL. It not only offers more holistic and in-depth insights into the temporal characteristics of SRL but also addresses, to some extent, the concerns of ontological flatness proposed by Reimann et al. (2014).

The current findings have important implications for the research and practice around SRL in the context of AOL. First, considering that the L-SRL preferred performance-avoidance goals and ignored time management and notetaking, instructors should encourage students to pursue mastery learning goals and actively engage in time management and notetaking, especially in AOCs. Additionally, this study informs the design of adaptive SRL interventions. Since not all learners were able to equally benefit from fixed SRL support, SRL interventions should be tailored to meet the needs of students with different patterns of SRL behaviors. We highly recommend that educators develop adaptive SRL interventions that can track and evaluate SRL behavior changes on the fly and provide immediate and personalized suggestions on SRL strategy use. Additionally, the temporal analyses of learners’ interactions with SRL support can evaluate how an SRL intervention relates to learning outcomes. Indeed, Damgaard and Nielsen (2018) highlighted the importance of examining the mechanisms that behavioral interventions affect, as interventions may fall short of intended positive effects if the understanding of the likely affected behavioral pathways is insufficient. Finally, the visualization of temporal SRL behaviors conveys quantitative information in a more digestible and actionable way, which enables instructors to (1) pinpoint how SRL processes unfold over time and differ across different SRL groups and (2) determine when and how to intervene as warranted.

The current study has some limitations that should be addressed in future research. First, all the participants were graduate students from universities located in northern Taiwan, which may limit the generalizability of our findings. Future studies should include a larger sample of students at other educational levels and from different countries/regions. Secondly, as with most SRL research, this study conducted a postanalysis of students’ SRL behaviors. Future studies could integrate this postanalysis into AOCs to offer students immediate learning analytics-based feedback to support their calibration for SRL. Thirdly, this study did not collect students’ scores of prior knowledge tests regarding research ethics, which limits the examination of the relationship between students’ prior knowledge and their SRL behavioral traces. In the future, researchers could investigate whether student groups with distinct SRL processes differ in prior knowledge and how students with different levels of prior knowledge perform their SRL behavioral trajectories. Fourthly, the participants’ SRL behaviors were dominated by time management due to the time restrictions imposed in the course, which may make our study not represent most behavioral data-based SRL studies, especially in authentic learning settings in which time management is usually in the background. Thus, we encourage researchers to examine further the association of time management with learning outcomes in AOL settings. For example, future studies could explore how students’ learning outcomes are related to the frequency of time management behaviors or SRL behavioral sequences involving time management. Moreover, it remains unclear whether our findings about distinct SRL behavioral patterns can be generalized to large-scale open AOL environments, such as MOOCs. Finally, because SRL is a multidimensional construct that includes (meta)cognitive, emotional, motivational, and behavioral components, it is difficult to use a single data source to capture the full range of SRL processes. Hence, future researchers could utilize multimodal multichannel data (e.g., physiological measures) to create a more comprehensive picture of SRL processes.

## Data availability statement

The raw data supporting the conclusions of this article will be made available by the authors, without undue reservation.

## Ethics statement

Ethical approval was not provided for this study on human participants because all of the research data were stored on the first author’s and the corresponding author’s personal computers that are password-protected, and can be accessed by only the first author and corresponding author of this paper for research purposes. All of the participants in the sample voluntarily participated in the study. Individual responses were confidential. No identifying information linked responses to individuals, and thus students’ private information was fully protected in the study. There are no conflicts of interest to declare. Written informed consent for participation was not required for this study in accordance with the national legislation and the institutional requirements.

## Author contributions

JS contributed to course development, data collection, research idea, and manuscript writing and editing. YL contributed to research idea, data analysis, and manuscript writing and revision. XL contributed to manuscript writing and editing. XH contributed to manuscript review and editing. All authors contributed to the article and approved the submitted version.

## Funding

This research was supported by the National Science and Technology Council (formerly Ministry of Science and Technology) in Taiwan through Grant numbers MOST 111-2410-H-A49-018-MY4, MOST 110-2511-H-A49-009-MY2, MOST 107-2628-H-009-004-MY3, MOST 105-2511-S-009-013-MY5, and NSC 99-2511-S009-006-MY3. The publication was made possible in part by support from the HKU Libraries Open Access Author Fund sponsored by the HKU Libraries.

## Conflict of interest

The authors declare that the research was conducted in the absence of any commercial or financial relationships that could be construed as a potential conflict of interest.

## Publisher’s note

All claims expressed in this article are solely those of the authors and do not necessarily represent those of their affiliated organizations, or those of the publisher, the editors and the reviewers. Any product that may be evaluated in this article, or claim that may be made by its manufacturer, is not guaranteed or endorsed by the publisher.
