# Supplementary material for: Temporal learning analytics to explore traces of self-regulated learning behaviors and their associations with learning performance, cognitive load, and student engagement in an asynchronous online course
Source: Front Psychol. 2023 Jan 23;13:1096337. doi: 10.3389/fpsyg.2022.1096337 (PMC9901299; doi:10.3389/fpsyg.2022.1096337)
Supplement: Supplementary file 1 [file Table_1.DOCX]

# Appendix A. The adjusted residuals (*z* scores) for the online SRL behaviors of the two groups

|  | Group | SU | G1 | G2 | G3 | SD | TN | CT | RD | TT | OT |
| --- | --- | --- | --- | --- | --- | --- | --- | --- | --- | --- | --- |
| SU | H-SRL | -0.281 | -1.314 | -2.130 | -2.616 | -1.199 | -3.723 | -10.128 | -0.865 | **31.420** | 0.501 |
|  | L-SRL | -5.715 | -1.499 | -3.837 | -6.026 | -1.958 | -4.717 | -5.841 | -1.440 | **29.913** | -2.206 |
| G1 | H-SRL | **15.336** | -0.437 | -0.709 | -0.870 | -1.229 | -1.422 | -5.802 | -0.520 | -1.155 | -0.849 |
|  | L-SRL | **6.967** | -0.303 | -0.775 | -1.217 | -1.534 | -1.095 | -2.093 | -0.291 | -0.566 | -0.770 |
| G2 | H-SRL | **24.856** | -0.709 | -1.149 | -1.411 | -1.991 | -2.304 | -9.404 | -0.843 | -1.872 | -1.376 |
|  | L-SRL | **19.094** | -0.775 | -1.983 | -3.114 | -3.613 | -2.804 | -5.358 | -0.744 | -3.234 | -1.971 |
| G3 | H-SRL | **30.523** | -0.870 | -1.411 | -1.732 | -2.445 | -2.830 | -11.548 | -1.035 | -2.299 | -1.690 |
|  | L-SRL | **30.905** | -1.217 | -3.114 | -4.891 | -6.167 | -4.403 | -8.414 | -1.169 | -5.607 | -3.094 |
| SD | H-SRL | -4.065 | **18.061** | **29.274** | **35.948** | -3.452 | -3.995 | -16.301 | -1.461 | -3.245 | -2.385 |
|  | L-SRL | -8.629 | **8.533** | **21.839** | **34.295** | -7.776 | -5.551 | -10.610 | -1.474 | -7.070 | -3.902 |
| TN | H-SRL | -4.451 | -1.422 | -2.304 | -2.830 | -2.536 | **31.945** | -9.839 | -1.691 | -3.755 | -0.720 |
|  | L-SRL | -5.511 | -1.095 | -2.804 | -4.403 | -2.535 | **30.615** | -3.343 | -0.008 | -5.047 | -1.557 |
| CT | H-SRL | -15.611 | -5.802 | -9.404 | -11.548 | -1.129 | -9.111 | **28.112** | **3.809** | -15.324 | **3.041** |
|  | L-SRL | -10.527 | -2.093 | -5.358 | -8.414 | 1.562 | -2.573 | **23.306** | **3.334** | -9.646 | **4.373** |
| RD | H-SRL | -1.721 | -0.520 | -0.843 | -1.035 | -0.721 | -1.038 | **3.440** | 1.039 | -1.373 | -1.009 |
|  | L-SRL | -1.635 | -0.291 | -0.744 | -1.169 | 0.138 | -1.052 | **4.670** | -0.279 | -1.340 | -0.739 |
| TT | H-SRL | -3.751 | -1.299 | -2.106 | -2.586 | 0.129 | -3.668 | **2.091** | -1.545 | **8.871** | 0.121 |
|  | L-SRL | -6.912 | -1.604 | -4.104 | -6.445 | **9.763** | -3.101 | **2.017** | 0.805 | **2.096** | **6.349** |
| OT | H-SRL | **3.225** | -0.849 | -1.376 | -1.690 | **18.882** | -2.352 | -7.803 | -1.009 | -0.777 | -1.001 |
|  | L-SRL | 0.388 | -0.770 | -1.971 | -3.094 | **12.850** | -2.376 | -2.965 | -0.739 | -3.548 | -0.842 |

*Notes*. H-SRL: high online self-regulated learning group; L-SRL: low online self-regulated learning group; SRL behavior abbreviations: SU = Selecting a learning unit, G1 = Choosing a mastery learning goal, G2 = Choosing a performance-approach learning goal, G3 = Choosing a performance-avoidance learning goal, SD = Setting a learning duration, TN = Taking notes, CT = Checking remaining learning time, RD = Resetting a learning duration, TT = Taking a unit test, OT = Performing off-task behaviors. All *z* scores greater than 1.96 are highlighted in bold.
